# Task dependent early landing kinematics after return to sport in individuals with ACL reconstruction: a secondary analysis

**DOI:** 10.1186/s12891-026-09967-8

**Published:** 2026-05-13

**Authors:** Bolun Wang, Ziyue Wang, Lin Cheng

**Affiliations:** 1 Department of Physical Education, Woosuk University, Wanju-gun, Jeollabuk-do Republic of Korea; 2 School of Nursing and Midwifery, University of Galway, Galway, Ireland; 3 Regenerative Medicine Institute (REMEDI), Biomedical Sciences Building. School of Medicine, National University of Ireland (NUI), Galway, Ireland

**Keywords:** ACLR, Knee internal rotation, Task dependency, Landing biomechanics

## Abstract

**Background/objective:**

Despite returning to sport (RTS), individuals after anterior cruciate ligament reconstruction (ACLR) may exhibit residual biomechanical alterations that are not fully captured by conventional RTS assessments. This study investigated 3D lower-limb kinematics during the critical early landing phase (0–100 ms) in individuals who had already returned to sport after ACLR, with the aim of examining how task-specific demands and localized acute fatigue influence early landing movement patterns.

**Methods:**

In this secondary analysis of a publicly available 3D motion capture dataset, 19 participants post ACLR (involved limb) and 22 healthy controls (dominant limb) were assessed. Knee, ankle, and pelvic joint angles were extracted at initial contact (IC, 0 ms) and at 25, 50, 75, and 100 ms intervals. Tasks included the unilateral counter movement jump (uCMJ) and single leg forward hop (SLH) under fatigued and non-fatigued conditions. Linear mixed-effects models compared kinematics across groups and conditions.

**Results:**

The ACLR group demonstrated a consistent transverse plane kinematic pattern suggestive of greater knee internal rotation during the early landing phase across all tasks (*p* < 0.05). Sagittal plane strategies showed marked task dependency. During the SLH task, the ACLR group showed a stiffer landing pattern, characterized by reduced knee flexion by 2°–4° and concurrent ankle dorsiflexion restriction around 50 ms after IC. Conversely, no significant between-group differences in knee flexion were observed during the uCMJ task. Furthermore, the localized acute fatigue protocol did not significantly alter these observed inter-group discrepancies.

**Conclusions:**

Individuals who had returned to sport after ACLR may retain residual early landing kinematic patterns, including a consistent transverse plane movement pattern and task specific stiff landing strategies. Future RTS assessment and post-RTS monitoring may benefit from placing greater emphasis on tasks with substantial horizontal braking demands, such as the SLH, as these tasks may provide additional information on residual early landing movement control patterns beyond conventional performance based outcomes. Rehabilitation may benefit from targeting landing quality, rotational control, and distal ankle buffering capacity.

**Supplementary Information:**

The online version contains supplementary material available at 10.1186/s12891-026-09967-8.

## Introduction

Anterior cruciate ligament injury is a common knee injury in athletic populations and is associated with substantial long-term functional consequences [1, 2]. Although ACL reconstruction combined with systematic rehabilitation can restore fundamental mechanical stability [3, 4], reinjury rates following return to sport remain persistently high [5, 6]. Current RTS assessments predominantly rely on time since surgery, static muscle strength symmetry, and functional performance measures [7, 8]. However, increasing evidence indicates that these macroscopic indicators may not fully capture residual biomechanical and movement control alterations in ACLR individuals during high-demand dynamic tasks [9, 10]. This discrepancy between conventional RTS assessment outcomes and actual sporting demands highlights the need to characterize residual biomechanical and movement control alterations that may persist after RTS, particularly during high demand sport like tasks [11, 12].

ACL injury mechanisms are highly time sensitive, with the vast majority of non contact injuries occurring during the very early phase following foot contact, typically within the first 100 ms after ground contact [13, 14]. During this critical time window, the lower limb joints must rapidly absorb ground reaction forces while maintaining dynamic stability; any delay or abnormality in neuromuscular control may result in excessive tensile loading of the ACL [12, 15]. Moreover, competitive sports are often characterized by prolonged and high intensity physical demands, and fatigue is widely recognized as an important external factor that can impair neuromuscular control, alter lower limb kinematic patterns, and potentially increase injury risk [16]. Therefore, systematically examining early post landing kinematics (0–100 ms) under both fatigued and non-fatigued conditions may help reveal residual movement control alterations that persist after ACLR and RTS.

Importantly, Individuals who have already returned to sport are a clinically relevant group. Medical clearance and successful RTS do not necessarily indicate complete biomechanical recovery. Therefore, examining landing mechanics in this population may help identify residual post-RTS movement control alterations. These findings may inform post clearance monitoring, late stage rehabilitation, and future prospective studies.

To investigate the aforementioned issues, this study conducted a secondary analysis of the comprehensive motion capture dataset published by Calisti et al. (2025) [17]. Although the original dataset contains six different jump tasks, this study specifically focused on a comparative analysis of the uCMJ and the SLH based on specific selection criteria. The rationale for this selection is threefold: First, compared to bilateral tasks (e.g., drop jumps), unilateral tasks eliminate the compensatory off loading mechanism of the non-injured limb, compelling the affected limb to independently manage landing impact, thereby potentially revealing residual movement control alterations [9]. Second, the original authors’ initial analysis confirmed that the SLH performed under fatigued conditions yielded the highest predictive accuracy in differentiating ACLR individuals from healthy controls, demonstrating high assessment sensitivity [17]. Third, the uCMJ and SLH represent two distinct loading modes: one dominated by vertical braking and the other incorporating a significant horizontal braking component, respectively [18, 19]. By contrasting these vertical and horizontal loading demands, this analysis provides a task dependent framework for examining how different landing constraints may reveal residual lower limb movement control alterations after ACLR.

Based on the above background, the present secondary analysis aimed to compare early landing lower limb kinematic characteristics between individuals who had already returned to sport after ACL reconstruction and healthy controls during the execution of uCMJ and SLH tasks under fatigued and non-fatigued conditions within the first 100 ms following foot contact. The analysis focused on three dimensional knee kinematics in the sagittal, frontal, and transverse planes, with concurrent examination of ankle and pelvic movement strategies. By characterizing task specific kinematic differences within this critical time window, the present study sought to identify residual movement control characteristics that remain incompletely restored after RTS. These findings may inform post clearance monitoring, targeted late stage rehabilitation, and future prospective research aimed at refining RTS assessment frameworks.

## Methods

### Study design and data source

This study is a secondary analysis of a publicly available biomechanics dataset originally published by Calisti et al. The data collection was approved by the Ethics Committee of the University of Innsbruck (Certificate No. 98/2022), and all participants provided written informed consent prior to participation.

The original dataset comprised 43 physically active individuals, including 21 participants with anterior cruciate ligament injury (ACL group) and 22 healthy controls (Control group). To minimize potential confounding factors and ensure homogeneity within the post–ACL reconstruction population, two participants who had received conservative treatment (ACLD) were excluded from the secondary analysis. Consequently, the final analytical sample consisted of 41 participants, including 19 individuals who had undergone ACL reconstruction (ACLR group) and 22 healthy controls (Control group).

Inclusion criteria for the ACLR group were unilateral ACL rupture treated with surgical reconstruction and medical clearance to return to level I sports (involving cutting, pivoting, and multidirectional jumping activities) for at least one year prior to testing. Control participants reported no history of lower extremity musculoskeletal injury within the six months preceding testing. For limb selection, the original dataset included bilateral measurements for all participants. In this secondary analysis, the involved, surgically reconstructed limb was selected in the ACLR group because it was the clinically relevant limb for evaluating post-reconstruction movement patterns. In the Control group, the dominant limb, defined as the preferred kicking leg, was selected as a standardized reference limb. This analytic choice was made to provide a clinically meaningful comparison between the reconstructed limb and a consistent reference limb in healthy controls.

The IKDC subjective knee form and ACL-RSI scale were collected as part of the original data collection protocol (Calisti et al., 2025) and completed by participants on the day of testing before the experimental tasks. These measures are reported here as descriptive characteristics of the cohort’s self reported knee function and psychological readiness at the time of assessment, rather than as primary or secondary outcome variables of the present analysis.

### Experimental protocol

All participants performed uCMJ and SLH tasks under both non-fatigued and fatigued conditions.

For the uCMJ task, participants stood on one leg at the center of the force plate with their hands placed on their hips, while the non-stance leg was maintained at approximately 90° of knee flexion. Participants performed a rapid countermovement to a self selected depth and then jumped vertically with maximal effort, landing on the same limb. For the SLH task, participants stood at a distance corresponding to 100% of their leg length, defined as the distance from the anterior superior iliac spine to the lateral malleolus, and performed a single-leg forward jump to land on the center of the force plate.

A trial was considered successful if participants maintained their hands on their hips after landing, preserved postural stability, and held the position of maximal knee flexion for at least 2 s. For each task and condition, three successful trials were collected for subsequent analysis.

### Fatigue protocol

Following the baseline (non-fatigued) measurements, subjects underwent a standardized localized functional fatigue protocol. This protocol consisted of multiple sets of single-leg squats (descending until the knee reached 90° of flexion to touch a box) and single-leg step-ups (30 cm step box). The onset of fatigue was confirmed based on the decrement in maximal vertical jump height and the Borg CR10 Scale of Perceived Exertion. Specifically, fatigue was defined as meeting either of the following criteria: (1) a reduction in maximal jump height of ≥ 20%, or (2) a reduction in maximal jump height of ≥ 10% coupled with a Borg CR10 score > 5. Immediately upon reaching the fatigued state, subjects proceeded to the post fatigue landing tasks. To ensure the continuous maintenance of fatigue throughout the testing session, subjects were required to perform additional sets of single-leg squats and step-ups after every two jump trials before continuing with the remaining assessments, in accordance with the established experimental procedures.

### Data acquisition and processing

Kinematic data were captured using a 10 camera three dimensional motion capture system (Vicon Motion Systems, Oxford, UK) at a sampling rate of 250 Hz. Ground reaction forces (GRF) were recorded synchronously using two force platforms (AMTI, USA; Kistler, Switzerland) sampled at 1000 Hz. This study utilized processed joint angle data directly provided by the original dataset authors. Briefly, raw marker trajectories and GRF data were filtered using a third order, zero phase lag, low pass Butterworth filter with a cut off frequency of 15 Hz. Inverse kinematics were calculated in OpenSim (v4.3) using a modified full body musculoskeletal model developed by Catelli et al. A pivotal feature of this model is the refined definition of the knee joint, which was assigned three degrees of freedom (3 DoF): flexion/extension, adduction/abduction, and internal/external rotation. This configuration allowed estimation of knee motion in the sagittal, frontal, and transverse planes during landing.

### Outcome measures and time window selection

Given that non-contact ACL injuries typically occur during the ultra early phase of ground contact, specifically within the first 100 ms, with the highest risk interval identified between 40 and 60 ms, the analysis window for this study was confined to the initial 100 ms of the landing phase to focus on the high risk period.

To characterize early weight acceptance kinematics while maintaining statistical interpretability and avoiding the redundancy of frame by frame testing, a discrete time point sampling strategy was adopted. Based on the original sampling frequency of 250 Hz, a 25 ms interval was used for data extraction. Therefore, five time points were selected: initial contact (IC, 0 ms), and 25, 50, 75, and 100 ms after IC. These time points were chosen to represent key phases of early landing: 0 ms reflects the posture at initial contact; 25–75 ms captures the early weight-acceptance phase during which ground reaction force and joint motion change rapidly, with 50 ms providing a clinically interpretable reference point within this high loading interval; and 100 ms represents the end of the ultra early landing window. IC was defined as the moment when the vertical ground reaction force (vGRF) first exceeded a 20 N threshold. The primary outcome variables included knee joint angles (flexion, adduction [where negative values represent valgus], and internal rotation), ankle flexion angle (dorsiflexion), and pelvic list (frontal plane movement).

### Statistical analysis

All statistical analyses were performed using the R software environment (R Foundation for Statistical Computing, Vienna, Austria). For baseline characteristics and demographic data, continuous variables are presented as mean ± standard deviation, while categorical variables are reported as frequencies (percentages). Inter-group comparisons were conducted using independent samples t-tests for continuous variables and Fisher’s exact test for gender distribution. The level of statistical significance was set at *p* < 0.05. Clinical variables applicable only to the ACLR group (e.g., time since injury, graft type, meniscectomy) were analyzed using descriptive statistics only.

For the kinematic characteristics during the landing phase (0–100 ms post initial contact), linear mixed-effects models were employed. To examine time point specific between group differences within the predefined early landing window, separate LMMs were constructed for each discrete time point (0, 25, 50, 75, and 100 ms). This modelling strategy was selected because it accommodates the repeated measures structure of the dataset, allows covariate-adjusted estimation, and provides clinically interpretable estimates at predefined early landing time points. Although continuous time-series approaches such as Statistical Parametric Mapping (SPM) may provide a more complete representation of temporal dynamics, the present LMM based approach was chosen to align with the predefined discrete time point design and the covariate-adjusted aims of this secondary analysis. In each model, “Subject ID” was included as a random intercept to account for the intrasubject correlation inherent in repeated measures. Fixed effects included Group (ACLR vs. Control), Fatigue State (Fatigued vs. Non-fatigued), and their interaction (Group × Fatigue). To strictly control for potential confounders, sex, age, and body mass index (BMI) were incorporated as covariates in all models.

Statistical results are reported as estimated marginal means (EMMs) and their standard errors (SE). The threshold for statistical significance was set at *p* < 0.05, with marginal significance defined as *p* < 0.10. Because the present analysis was exploratory and focused on predefined clinically interpretable time points within the early landing window, multiple comparison corrections were not applied across time points. We acknowledge that this approach may increase the risk of type I error. Therefore, statistically significant findings were interpreted cautiously, with emphasis placed on consistency in effect direction across adjacent time points and temporal alignment with key early landing mechanical events rather than isolated single time point significance.

## Results

### Baseline characteristics of the participants

A total of 41 participants were included in the final analysis, comprising 19 individuals in the ACLR group and 22 in the Control group. No statistically significant differences were observed between the two groups in terms of age, height, body mass, body mass index (BMI), or physical activity level (all *p* > 0.05), indicating well balanced baseline characteristics between groups (Table 1). The mean time since surgery in the ACLR group was 3.84 ± 2.61 years. The most common graft type was hamstring tendon autograft (*n* = 12, 63.2%), followed by quadriceps tendon autograft (*n* = 4, 21.1%) and bone–patellar tendon–bone autograft (*n* = 3, 15.8%). Additionally, 47.4% (*n* = 9) of participants in the ACLR group had a history of meniscectomy. Regarding self reported knee function, the ACLR group demonstrated a mean IKDC [20] score of 92.50 ± 7.95%, reflecting good subjective knee function. The mean ACL RSI [21] score was 65.86 ± 18.55, and the Tampa Scale of Kinesiophobia score was 21.21 ± 5.02.

**Table 1:** Baseline characteristics of the study participants

Variable	ACLR (*n* = 19)	Control (*n* = 22)	*p* value
Age (years)	24.37 ± 3.64	26.45 ± 3.20	0.058
Height (m)	1.75 ± 0.09	1.73 ± 0.11	0.612
Weight (kg)	69.34 ± 11.00	68.05 ± 14.81	0.755
BMI (kg/m²)	22.56 ± 1.67	22.45 ± 2.54	0.872
Sex, n (%)			1.000
Female	10 (52.6)	12 (54.5)	
Male	9 (47.4)	10 (45.5)	
Time from injury (years)	3.84 ± 2.61	NA	NA
Graft type, n (%)			NA
PT	3 (15.8)	NA	
QT	4 (21.1)	NA	
STG	12 (63.2)	NA	
Meniscectomy, n (%)			
Yes	9 (47.4)	NA	
No	10 (52.6)	NA	
Physical activity (days/week)	4.74 ± 1.27	4.59 ± 1.12	0.698
IKDC subjective score (%)	92.50 ± 7.95	NA	NA
ACL-RSI score	65.86 ± 18.55	NA	NA
Tampa scale of kinesiophobia	21.21 ± 5.02	NA	NA

Values are presented as mean ± standard deviation or number (percentage)

*ACLR* Anterior cruciate ligament reconstruction, *BMI* Body mass index, *IKDC* International Knee Documentation Committee, *ACL-RSI* Anterior Cruciate Ligament–Return to Sport after Injury scale

### Kinematic characteristics during single-leg forward hop landing

During the landing phase of the SLH task (0–100 ms), the ACLR group exhibited task specific sagittal plane stiffening and a consistent transverse plane kinematic pattern suggestive of greater knee internal rotation. No significant Group × Fatigue interaction effects were observed for any analyzed variables (*p* > 0.17), indicating that the localized acute fatigue protocol used in this study did not significantly modify the between-group kinematic differences. (Table 2 and Supplementary Table S1).

**Table 2:** Estimated marginal means of lower-limb joint angles during single-leg forward hop (SLH) landings, by group and fatigue condition

Result Category	Joint Angle	Time from IC (ms)	ACLR –F(°)	ACLR – NF(°)	Control – F(°)	Control – NF(°)	Group *p*-value
Primary	Knee Flexion	0	19.6(1.3)	20.5(1.3)	19.7(1.2)	20.3(1.2)	0.98
Primary	Knee Flexion	25	25.6 (1.1)	26.8 (1.1)	27.0 (1.1)	27.2 (1.1)	0.38
Primary	Knee Flexion	50	40.2 (1.4)	40.5 (1.4)	44.3 (1.3)	42.4 (1.3)	0.04*
Primary	Knee Flexion	75	51.4 (1.3)	51.4 (1.3)	54.4 (1.2)	53.1 (1.2)	0.09†
Primary	Knee Flexion	100	58.1 (1.1)	58.2 (1.1)	60.4 (1.0)	59.9 (1.0)	0.13
Primary	Knee Rotation	0	6.9 (2.1)	6.7 (2.1)	1.3 (1.9)	0.6 (1.9)	0.06†
Primary	Knee Rotation	25	8.0 (2.2)	7.6 (2.2)	0.8(2.0)	0.4 (2.0)	0.02*
Primary	Knee Rotation	50	7.9 (1.9)	7.6 (1.9)	2.2 (1.7)	1.6 (1.7)	0.04*
Primary	Knee Rotation	75	7.0 (1.8)	7.2 (1.8)	2.3 (1.7)	1.9 (1.7)	0.06†
Primary	Knee Rotation	100	7.5 (1.8)	7.1 (1.8)	2.6 (1.7)	2.2 (1.7)	0.06†
Primary	Ankle Dorsiflexion	0	-17.9 (4.0)	-19.0 (4.0)	-6.7 (3.7)	-9.1 (3.7)	0.05†
Primary	Ankle Dorsiflexion	25	-13.1 (1.7)	-12.7 (1.7)	-6.9 (1.6)	-7.0 (1.6)	0.01*
Primary	Ankle Dorsiflexion	50	-2.3 (1.0)	-2.7 (1.0)	0.3 (1.0)	-0.2 (1.0)	0.08†
Primary	Ankle Dorsiflexion	75	1.6 (1.0)	1.1 (1.0)	4.6 (1.0)	4.2 (1.0)	0.04*
Primary	Ankle Dorsiflexion	100	5.8 (1.1)	5.4 (1.1)	9.3 (1.0)	9.0 (1.0)	0.02*
Secondary	Knee Adduction	0	1.9 (0.5)	1.6 (0.5)	1.5 (0.4)	1.3 (0.4)	0.49
Secondary	Knee Adduction	25	1.9 (0.5)	1.6 (0.5)	1.9 (0.5)	1.8 (0.5)	0.96
Secondary	Knee Adduction	50	-0.5 (0.7)	-0.6 (0.7)	0.1 (0.6)	0.6 (0.6)	0.49
Secondary	Knee Adduction	75	-1.3 (0.8)	-1.6 (0.8)	-0.1 (0.8)	0 (0.8)	0.28
Secondary	Knee Adduction	100	-1.5 (0.9)	-1.7 (0.9)	-1.1 (0.8)	-0.7 (0.8)	0.73
Secondary	Pelvic Drop	0	15.5 (0.9)	15.0 (0.9)	14.1 (0.8)	13.8 (0.8)	0.28
Secondary	Pelvic Drop	25	14.3 (0.8)	13.9 (0.8)	12.8 (0.8)	12.7 (0.8)	0.20
Secondary	Pelvic Drop	50	10.8 (0.8)	10.8 (0.8)	9.2 (0.8)	9.5 (0.8)	0.19
Secondary	Pelvic Drop	75	8.2 (0.9)	8.3 (0.9)	7.0 (0.8)	7.0 (0.8)	0.35
Secondary	Pelvic Drop	100	6.9 (0.9)	6.9 (0.9)	5.6 (0.8)	5.3 (0.8)	0.29

*F* Fatigue condition, *NF* Non-Fatigue condition

Values are estimated marginal means (EMMs), with standard errors (SE) in parentheses. All joint angles are reported in degrees (°). Group p-values reflect the main effect of group (ACLR vs. Control) from linear mixed-effects models adjusted for sex, age, and BMI

Time points (0–100 ms) are relative to initial contact (IC), with 0 ms representing the moment of IC and subsequent values indicating milliseconds after IC

Variables are classified as Primary (showing statistically significant or clinically meaningful group differences at multiple time points) or Secondary (supporting variables without consistent significant findings)

Significance is denoted as: *p < 0.05, †*p* < 0.10

Angle conventions: Negative values for ankle dorsiflexion indicate plantarflexion ; negative values for knee adduction indicate knee abduction ; pelvic drop values reflect frontal plane pelvic tilt relative to the contralateral side

In the sagittal plane, the ACLR group demonstrated a distinct stiff landing strategy, characterized by concomitant limitations in knee and ankle joint excursions. Although no significant between group differences in knee flexion angle were observed at initial contact (IC), the ACLR group exhibited significantly smaller knee flexion angles during the subsequent weight acceptance phase. This difference reached statistical significance at 50 ms after IC (*p* = 0.04), with the ACLR group showing approximately 2°–4° less knee flexion than the control group (e.g., under fatigued conditions: ACLR 40.2° ± 1.4° vs. Control 44.3° ± 1.3°). A similar trend toward reduced knee flexion was also observed at 75 ms (*p* = 0.09). Consistent with the stiff knee pattern, the ACLR group exhibited significantly reduced ankle dorsiflexion (i.e., greater plantarflexion) throughout the early landing phase. Intergroup differences reached significance at 25 ms (*p* = 0.01), 75 ms (*p* = 0.04), and 100 ms (*p* = 0.02) post IC. Notably, during the ultra early phase (25 ms), the ACLR group displayed a more plantarflexed position (e.g., under fatigued conditions: −13.1° vs. −6.9°), suggesting a limited capacity for shock absorption via the ankle joint.

In the transverse plane, the ACLR group displayed a group-level pattern suggestive of greater knee internal rotation. At 25 ms (*p* = 0.02) and 50 ms (*p* = 0.04) post IC, the ACLR group exhibited significantly greater knee internal rotation compared to the Control group. Specifically, the ACLR group maintained internal rotation of approximately 7°–8°, whereas the Control group remained near a neutral position (approximately 0.8°–2.2°). This difference was concentrated within the first 50 ms of landing, suggesting altered rotational control strategies during early impact absorption.

In contrast, frontal plane kinematics were comparable between groups; no statistically significant differences were found for knee adduction or pelvic drop at any time point (all *p* > 0.19).

### Unilateral countermovement jump landing kinematics

Consistent with the findings from the SLH task, no significant group × fatigue interaction effects were observed for any of the analyzed variables during the uCMJ task (all *p* > 0.17), indicating that the localized acute fatigue protocol used in this study did not significantly modify the between group kinematic differences (Table 3 and Supplementary Table S2). Overall, during the landing phase of the uCMJ, the ACLR group exhibited restricted distal joint (ankle) motion and a consistent transverse-plane kinematic pattern suggestive of greater knee internal rotation, while no significant deficits in proximal (knee) flexion magnitude were observed.

**Table 3:** Estimated marginal means of lower-limb joint angles during unilateral countermovement jump (uCMJ) landings, by group and fatigue condition

Result Category	Joint Angle	Time from IC (ms)	ACLR –F(°)	ACLR – NF(°)	Control – F(°)	Control – NF(°)	Group *p*-value
Primary	Knee Flexion	0	21.7(1.2)	22.7(1.2)	21.1(1.1)	21.3(1.1)	0.70
Primary	Knee Flexion	25	25.7 (1.0)	26.7 (1.0)	26.3 (1.0)	26.9(1.0)	0.69
Primary	Knee Flexion	50	31.7 (1.4)	32.7 (1.4)	32.7 (1.3)	34.1 (1.3)	0.60
Primary	Knee Flexion	75	39.3 (1.7)	40.6 (1.7)	41.2 (1.6)	42.9 (1.6)	0.44
Primary	Knee Flexion	100	48.7 (1.9)	50.4 (1.9)	52.3 (1.8)	52.6 (1.8)	0.19
Primary	Knee Rotation	0	3.8 (2.0)	4.2 (2.0)	-4.0 (1.8)	-3.5 (1.8)	0.01*
Primary	Knee Rotation	25	7.7 (2.0)	7.7 (2.0)	0.3 (1.8)	0.5 (1.8)	0.01*
Primary	Knee Rotation	50	9.0 (2.0)	9.0 (2.0)	2.6 (1.8)	2.4 (1.8)	0.02*
Primary	Knee Rotation	75	9.7 (1.9)	9.4 (1.9)	3.4 (1.8)	3.4 (1.8)	0.02*
Primary	Knee Rotation	100	9.5 (1.9)	8.9 (1.9)	3.2 (1.7)	3.2 (1.7)	0.02*
Primary	Ankle Dorsiflexion	0	-26.8 (3.2)	-26.3 (3.2)	-19.3 (3.0)	-21.3 (3.0)	0.09†
Primary	Ankle Dorsiflexion	25	-12.4 (3.0)	-12.3 (3.0)	-5.3 (2.7)	-6.6 (2.7)	0.09†
Primary	Ankle Dorsiflexion	50	3.1 (2.2)	3.3 (2.2)	8.6 (2.0)	8.2 (2.0)	0.07†
Primary	Ankle Dorsiflexion	75	12.2 (1.6)	12.1 (1.6)	16.7 (1.5)	16.4(1.5)	0.04*
Primary	Ankle Dorsiflexion	100	17.3 (1.2)	16.8 (1.2)	20.5 (1.1)	21.0 (1.1)	0.06†
Secondary	Knee Adduction	0	1.2 (0.4)	0.9 (0.4)	1.1 (0.4)	1.1 (0.4)	0.96
Secondary	Knee Adduction	25	1.7 (0.4)	1.6 (0.4)	1.8 (0.4)	2.0 (0.4)	0.91
Secondary	Knee Adduction	50	1.4 (0.5)	1.1 (0.5)	1.7 (0.5)	2.0 (0.5)	0.68
Secondary	Knee Adduction	75	0.2 (0.6)	-0.3 (0.6)	0.9 (0.6)	1.0 (0.6)	0.39
Secondary	Knee Adduction	100	-1.1 (0.8)	-1.2 (0.8)	0 (0.8)	0.01(0.8)	0.34
Secondary	Pelvic Drop	0	16.7 (1.1)	16.8 (1.1)	16.4 (1.0)	16.2 (1.0)	0.82
Secondary	Pelvic Drop	25	16.4 (1.1)	16.4 (1.1)	16.0 (1.0)	15.5 (1.0)	0.82
Secondary	Pelvic Drop	50	15.0 (1.1)	15.1 (1.1)	14.6 (1.1)	13.7 (1.1)	0.80
Secondary	Pelvic Drop	75	12.6 (1.2)	12.7 (1.2)	11.5 (1.1)	10.8 (1.1)	0.54
Secondary	Pelvic Drop	100	10.2 (1.2)	10.5 (1.2)	8.7 (1.2)	8.2 (1.2)	0.37

*F* Fatigue condition, *NF* Non-Fatigue condition

Values are estimated marginal means (EMMs), with standard errors (SE) in parentheses. All joint angles are reported in degrees (°). Group p-values reflect the main effect of group (ACLR vs. Control) from linear mixed-effects models adjusted for sex, age, and BMI

Time points (0–100 ms) are relative to initial contact (IC), with 0 ms representing the moment of IC and subsequent values indicating milliseconds after IC

Variables are classified as Primary (showing statistically significant or clinically meaningful group differences at multiple time points) or Secondary (supporting variables without consistent significant findings)

Significance is denoted as: *p < 0.05, †*p* < 0.10

Angle conventions: Negative values for ankle dorsiflexion indicate plantarflexion ; negative values for knee adduction indicate knee abduction ; pelvic drop values reflect frontal plane pelvic tilt relative to the contralateral side

In the sagittal plane, the ACLR group adopted a landing strategy that differed slightly from that observed during the SLH task. Although ankle joint motion remained restricted, knee flexion did not demonstrate a pronounced stiff landing pattern. Specifically, ankle dorsiflexion angles were consistently smaller in the ACLR group compared with the control group throughout the early landing phase, with the between group difference reaching statistical significance at 75 ms after initial contact (IC) (*p* = 0.04; ACLR 12.2° ± 1.6° vs. Control 16.7° ± 1.5°). At the remaining time points, this difference showed a trend toward significance (*p* = 0.06–0.09). In contrast to the restricted ankle motion, no significant between group differences in knee flexion angle were observed at any time point between 0 and 100 ms after IC (p range: 0.19–0.70), suggesting that during a vertical jumping task, individuals with ACL reconstruction may retain the capacity to attenuate landing impact through knee flexion.

In the transverse plane, the ACLR group demonstrated a consistent group-level pattern of greater knee internal rotation. Linear mixed-effects modeling revealed that knee internal rotation angles were significantly greater in the ACLR group than in the control group across all analyzed time points (0–100 ms; *p* = 0.01–0.02). Notably, this difference was already evident at initial contact, where the ACLR group exhibited a more internally rotated estimated knee position (e.g., under fatigued conditions: 3.8° ± 2.0°), whereas the control group tended toward an externally rotated or near neutral position (− 4.0° ± 1.8°). This kinematic offset persisted throughout the early landing phase, indicating a consistent pattern of altered transverse-plane rotational control during vertical landing tasks in individuals with ACL reconstruction.

In the frontal plane, no statistically significant between group differences were observed in knee adduction angle or pelvic drop at any analyzed time point (all *p* > 0.34). These findings suggest that during the uCMJ task, the ACLR group was able to maintain frontal plane postural control comparable to that of healthy controls.

## Discussion

The purpose of this study was to determine whether individuals who had already returned to sport following ACL reconstruction exhibit residual lower limb movement control alterations when performing jump–landing tasks with different loading demands under both fatigued and non-fatigued conditions after returning to sport. By analyzing the critical time window of 0–100 ms following foot contact, we found that although the ACLR group demonstrated frontal plane knee and pelvic kinematics comparable to healthy controls, group level kinematic differences were observed in the transverse and sagittal planes. Specifically, the ACLR group exhibited a consistent transverse plane kinematic pattern suggestive of greater knee internal rotation, task specific reductions in knee flexion during the SLH task, and restricted distal ankle dorsiflexion. These findings indicate that individuals who had already returned to sport after ACLR may continue to demonstrate residual early landing movement control alterations, and that these differences were not significantly modified by the localized acute fatigue protocol used in this study.

### Transverse-plane kinematics: a consistent knee internal rotation pattern

A notable finding of this study was the consistent pattern of greater knee internal rotation (IR) in the ACLR group during the early landing phase. Specifically, in the uCMJ task, this transverse plane difference was evident at initial contact (IC, 0 ms). In the SLH task, which involved greater horizontal momentum, a similar pattern emerged during the early weight acceptance phase and reached statistical significance within the first 25 ms.

While conventional clinical assessments and rehabilitation protocols often prioritize the correction of frontal plane stability (e.g., knee valgus) [12, 22], our findings suggest that knee adduction and pelvic control in the ACLR group were comparable to controls; this frontal plane recovery may coexist with residual transverse-plane kinematic differences. Notably, because these differences were already present at initial contact, they may not be solely caused by the impact after landing. They may reflect differences in landing preparation or early landing control. Such patterns may represent a compensatory movement strategy aimed at managing perceived joint instability [23].

In vitro biomechanical studies have established that excessive tibial rotation may increase strain on the ACL graft [24, 25]. Consequently, these persistent early-phase kinematic alterations may represent residual movement control adaptations that could contribute to the elevated risk of non-contact reinjury in individuals who have returned to sport following ACL reconstruction. However, this interpretation remains hypothetical. Kinematic data alone cannot confirm neuromuscular preactivation or feed-forward motor planning, nor can they exclude alternative explanations such as passive kinematic coupling driven by pelvic or trunk positioning or measurement uncertainty in skin-marker-based transverse plane knee kinematics. These findings therefore require further validation using joint kinetics, EMG, higher fidelity kinematic methods, and prospective study designs.

### Sagittal plane strategies: task dependency and landing stiffness

In contrast to the transverse-plane kinematic pattern described above, sagittal plane characteristics exhibited a clear task dependent pattern. During the uCMJ task, no significant between group differences in knee flexion angle were detected, suggesting that under primarily vertical braking conditions, sagittal plane knee flexion during early landing was broadly comparable between groups. However, during the SLH task, which imposes greater horizontal braking demands, individuals in the ACLR group demonstrated significantly reduced knee flexion at 50 ms after initial contact.

This time point falls within the early high-loading phase of landing, when ground reaction forces are rapidly increasing [13, 26]. Individuals after ACLR may momentarily limit knee flexion during this interval, a pattern consistent with previously reported reductions in knee flexion during hop landing after ACLR [27, 28]. This may reflect a task specific stiff landing strategy or a protective movement pattern during high demand braking. As loading demands decrease in the later stages of landing after 75 ms, this transient stiffening pattern gradually subsides, and joint kinematics return to levels comparable to controls.

Nevertheless, this stiffness for stability strategy may represent a potential biomechanical trade off. During the critical 50 ms loading window, restricted knee flexion may reduce the dynamic shock absorbing capacity of the lower limb [29]. This localized stiffness may reduce muscular buffering and increase reliance on intra-articular passive structures [30]. Consequently, this transient protective compensation may increase mechanical demands on passive knee structures during peak loading. Without inverse dynamics or prospective injury data, this finding should not be interpreted as direct evidence of increased graft loading or re-injury risk. Future studies incorporating joint moments, joint power absorption, and prospective follow up are needed to determine the mechanical relevance of this stiff-landing pattern.

### Kinetic chain effects: task specific manifestations of ankle buffering limitations

Beyond knee-level differences, this study observed restricted ankle dorsiflexion in the ACLR group, with task specific timing and magnitude.

In the vertically dominant uCMJ task, between group differences in ankle dorsiflexion were not statistically significant during the early phase but reached significance at 75 ms. This suggests that, under primarily vertical braking demands, ankle dorsiflexion restriction may become more apparent as weight acceptance progresses.

In contrast, in the SLH task, which involved greater horizontal momentum, this restriction presented as an early-onset and sustained pattern. The ACLR group showed signs of deviation at the initial contact (0 ms, *p* = 0.05), which rapidly evolved into a significant dorsiflexion deficiency within 25 ms. Despite a brief marginal transition at 50 ms, the between-group difference increased again during the late landing phase (75–100 ms). According to kinetic chain theory, the ankle joint serves as the primary line of defense for energy dissipation during landing [31]. The early onset of this distal restriction in SLH (at 25 ms) may promote the proximal transmission of impact loads [32]. This temporal sequence: ankle dorsiflexion restriction at 25 ms preceding the knee flexion reduction observed at 50 ms (Sect. 4.2), is consistent with a proximal-distal kinetic chain relationship, although direct causal evidence would require synchronised kinetic and EMG data.

Furthermore, this distal restriction provides may provide one possible biomechanical explanation for the transverse plane kinematic pattern observed in Sect. 4.1. In closed kinetic chain movements, restricted ankle dorsiflexion may compel the lower extremity to increase tibial internal rotation to compensate for the spatial demands of center of mass deceleration [33]. Consequently, the persistent transverse-plane kinematic pattern observed in the ACLR group may be multifactorial, potentially reflecting both altered anticipatory movement control strategies and compensatory movement adaptations driven by restricted ankle mobility. However, this proposed kinetic chain explanation remains inferential and should be confirmed in future studies using kinetic analyses, trunk and hip kinematics, and higher-fidelity assessment of transverse plane knee motion.

### Interpretation of fatigue effects

No significant Group × Fatigue interaction effects were observed for any of the analyzed variables. These findings suggest that the kinematic alterations observed in the ACLR group, including increased knee internal rotation and a stiffer landing strategy, may reflect relatively stable residual movement control patterns that were not further amplified by the localized acute muscular fatigue protocol used in this study.

However, fatigue in real sporting environments is multidimensional and may include peripheral muscular fatigue, cardiopulmonary or metabolic load, central fatigue, cognitive fatigue, reactive decision making demands, and the combined effects of repeated multidirectional movements. Localized peripheral fatigue primarily reflects fatigue of specific lower limb muscle groups, whereas central or mental fatigue may affect attention, decision making, motor planning, and neuromuscular coordination. Mixed physical cognitive fatigue may therefore impose broader constraints on landing and cutting mechanics than localized muscular fatigue alone. Previous studies have suggested that mental fatigue can alter drop landing motion control in individuals with functional ankle instability and that mixed physical mental fatigue can influence knee biomechanics and muscle activation during sidestep cutting in elite soccer players [34, 35].

Therefore, although the localized fatigue model used in the present study has advantages in terms of repeatability and experimental control, it may underestimate the fatigue related neuromuscular disruption that occurs in competitive sporting environments. Future studies should adopt more sport specific and multidimensional fatigue paradigms, such as whole body exercise protocols, repeated sprinting or cutting tasks, reactive deceleration tasks, mental fatigue induction, or physical cognitive dual task designs, to more accurately simulate the fatigue states associated with non-contact secondary ACL injury risk.

### Clinical translation of the present findings

The present findings suggest that tasks incorporating greater horizontal braking demands, such as the SLH, may be more sensitive to residual movement control patterns after ACLR than vertically dominant tasks such as the uCMJ. Given that RTS assessment pathways are not fully standardized across settings, the SLH may be considered as an additional task in settings that do not yet adequately incorporate horizontally demanding tasks or landing quality assessment [7]. Importantly, because the present cohort had already returned to sport, these findings should be interpreted as informing post-RTS monitoring and late stage rehabilitation rather than serving as direct evidence for pre-RTS clearance decisions.

For settings already using the SLH, assessment should extend beyond hop distance, completion time, or limb symmetry indices. The clinical relevance of the present findings should not be judged solely by the absolute magnitude of a single angular difference at one time point. Rather, these differences should be interpreted in relation to their consistency across time points, task demands, and inter-joint coordination. Although some angular differences were modest, previous biomechanical research suggests that landing kinematics of similar magnitude can carry injury relevant significance, particularly when they occur during early high-loading phases and are accompanied by coordinated changes across joints [12]. Reduced knee flexion and restricted ankle dorsiflexion may together indicate a stiffer landing strategy [32, 36], which in clinical practice may appear as reduced shock absorption, impaired knee ankle coordination, reduced post landing stability, or visibly stiff landing mechanics.

To address the short early landing time window, high-frame-rate smartphone video, slow motion playback, and frame-by-frame analysis software such as Kinovea [37] may help clinicians evaluate landing quality more systematically. For transverse plane rotational control, which is difficult to assess visually, portable IMU systems may provide a promising and more accessible adjunct to laboratory motion capture [38], although future research should determine whether these tools can reliably detect the movement features identified in the present study.

Beyond the choice of assessment tools, the ecological validity of the testing context itself also warrants consideration. Although tasks such as the SLH may provide a more mechanically demanding assessment of early landing control, they still represent relatively controlled and pre-planned laboratory tasks. Real sporting landings often occur under cognitive and perceptual demands, including anticipation, reaction, divided attention, and rapid decision making. Recent evidence suggests that mental fatigue and increased cognitive demands can alter single leg landing biomechanics and ACL risk related movement patterns [39, 40]. Therefore, future RTS assessments should progress beyond isolated movement tasks toward more ecologically valid paradigms that combine mechanical demands with cognitive load, reactive decision making, or dual task conditions. In this context, the SLH should be viewed as one component of a broader RTS assessment framework rather than a stand alone solution.

### Limitations

Although the present study provides novel evidence for understanding residual movement control patterns in individuals who have already returned to sport, several limitations should be acknowledged.

First, regarding limb selection, the dominant limb was selected in the control group as a standardised reference baseline, while the involved limb was analysed in the ACLR group, of which approximately 57% were non-dominant limbs. Although existing evidence suggests that between-limb kinematic differences during SLH tasks are generally small in healthy individuals [41], In addition, inter-limb asymmetries attributable to limb dominance have been reported to be task-specific and inconsistent in both magnitude and direction across individuals and tests [42]. the potential confounding influence of limb dominance cannot be entirely ruled out. Future studies should either prospectively match limb dominance between groups or conduct sensitivity analyses using the contralateral limb data available in the original dataset to quantify the magnitude of this potential confound.

Second, this study relied primarily on kinematic indicators. The lack of synchronized kinetic and electromyographical (EMG) data limits direct interpretation of joint loading, joint moment modulation, muscle activation timing, and neuromuscular pre-activation mechanisms. Therefore, the observed movement patterns should not be interpreted as direct evidence of ACL loading, feed forward motor control errors, or reinjury mechanisms.

Third, transverse plane knee kinematics derived from skin-marker-based motion capture are subject to known reliability and validity limitations. This issue is particularly relevant during high impact landing tasks, where soft tissue artefact may be substantial. Although inverse kinematics and the full body modelling approach used in the original dataset may improve tracking consistency, they do not eliminate soft tissue artefact. Therefore, the knee rotation findings should be interpreted as estimated transverse plane kinematic patterns rather than precise measures of tibiofemoral rotation. Future studies should validate these findings using higher fidelity methods such as biplanar fluoroscopy, bone anchored markers, cluster based tracking with soft tissue artefact compensation, or model based artefact correction.

Fourth, although the focus on the 0–100 ms high risk window and the use of predefined discrete time points enhanced clinical interpretability and facilitated covariate-adjusted modelling, this approach may have missed transient peaks, valleys, or short duration group differences occurring between sampled time points. This limitation is particularly relevant during the early landing phase, when joint kinematics and loading conditions can change rapidly and non-linearly. Continuous time series approaches, such as Statistical Parametric Mapping (SPM) or functional data analysis, should therefore be considered in future studies to validate and extend the present findings.

Fifth, formal multiple comparison correction was not applied across time points and outcome variables. This may increase the risk of type I error. To reduce the influence of isolated false positive findings, statistically significant results were interpreted in the context of consistency in effect direction across adjacent time points and temporal alignment with key early landing mechanical events. Nevertheless, the findings should be interpreted cautiously and considered exploratory.

Sixth, the localized peripheral fatigue protocol used in the original dataset, although standardized and reproducible, may not fully replicate the multidimensional fatigue states encountered in competitive sport. Therefore, the absence of a significant Group × Fatigue interaction should be interpreted within the context of this specific fatigue model.

Seventh, as this study is a secondary analysis of a publicly available dataset, it was constrained by the original experimental design and available variables; Certain key variables, such as joint torques, muscle synergy patterns, trunk control, cognitive load, and reactive decision making components, could not be incorporated into the analysis.

Finally, as a cross sectional investigation of individuals who had already returned to sport, the movement patterns identified in this study cannot be interpreted as causally related to future reinjury riskor as direct evidence for pre-RTS clearance decisions. Prospective longitudinal studies are required to determine whether these early landing kinematic patterns are present before RTS and whether they are associated with subsequent ACL injury.

## Conclusion

In summary, individuals who had already returned to sport after ACLR exhibited residual early landing kinematic patterns despite apparently restored frontal plane control. These patterns included a consistent transverse plane movement pattern suggestive of greater estimated knee internal rotation, restricted ankle dorsiflexion, and task specific sagittal plane differences. Specifically, knee flexion was largely comparable during the vertically dominant uCMJ task, whereas reduced knee flexion emerged during the horizontally demanding SLH task. These findings should be interpreted as exploratory movement control patterns rather than direct evidence of feed-forward motor control errors, graft loading, or reinjury mechanisms. Future RTS assessment frameworks and post-RTS monitoring may benefit from greater emphasis on tasks with horizontal braking demands, such as the SLH, because they may provide complementary information beyond primarily vertical tasks. Rehabilitation may benefit from targeting landing quality, rotational control, and distal ankle buffering capacity. Future prospective studies incorporating kinetic and EMG measures are needed to determine whether these patterns are associated with subsequent ACL injury risk.

## Supplementary Information

Supplementary Material 1.

## Data Availability

The data supporting the findings of this study are derived from a publicly available dataset originally published by Calisti et al. (2025) and can be accessed via PMID: 41093902.

## Abbreviations

uCMJ: unilateral counter movement jump
SLH: Single leg forward hop
RTS: Returning to sport
ACLR: Anterior cruciate ligament reconstruction
GRF: Ground reaction forces
EMG: Electromyographical
STA: Soft tissue artifacts
SPM: Statistical Parametric Mapping
IR: Internal rotation
IC: Initial contact
BMI: Body mass index
EMMs: Estimated marginal means
SE: Standard errors
