# Supplementary material for: Task dependent early landing kinematics after return to sport in individuals with ACL reconstruction: a secondary analysis
Source: BMC Musculoskelet Disord. 2026 May 13;27:589. doi: 10.1186/s12891-026-09967-8 (PMC13343948; doi:10.1186/s12891-026-09967-8)
Supplement: Supplementary file 1 — Supplementary Material 1. [file 12891_2026_9967_MOESM1_ESM.docx]

**Table S1.** P-values for the main effect of Condition and the Group × Condition interaction at each time point during single-leg forward hop (SLH) landings, from linear mixed-effects models.

| **Joint Angle** | **Time from IC (ms)** | **p (Condition)** | ****p (Group × Condition)**** |
| --- | --- | --- | --- |
| Knee Flexion | 0 | 0.21 | 0.81 |
| Knee Flexion | 25 | 0.09 | 0.32 |
| Knee Flexion | 50 | 0.79 | 0.17 |
| Knee Flexion | 75 | 0.99 | 0.38 |
| Knee Flexion | 100 | 0.85 | 0.51 |
| Knee Rotation | 0 | 0.81 | 0.56 |
| Knee Rotation | 25 | 0.57 | 0.93 |
| Knee Rotation | 50 | 0.62 | 0.76 |
| Knee Rotation | 75 | 0.77 | 0.50 |
| Knee Rotation | 100 | 0.54 | 0.82 |
| Ankle Dorsiflexion | 0 | 0.64 | 0.68 |
| Ankle Dorsiflexion | 25 | 0.70 | 0.69 |
| Ankle Dorsiflexion | 50 | 0.54 | 0.93 |
| Ankle Dorsiflexion | 75 | 0.38 | 0.92 |
| Ankle Dorsiflexion | 100 | 0.53 | 0.92 |
| Knee Adduction | 0 | 0.19 | 0.80 |
| Knee Adduction | 25 | 0.21 | 0.46 |
| Knee Adduction | 50 | 0.80 | 0.23 |
| Knee Adduction | 75 | 0.36 | 0.41 |
| Knee Adduction | 100 | 0.62 | 0.33 |
| Pelvic Drop | 0 | 0.31 | 0.76 |
| Pelvic Drop | 25 | 0.40 | 0.61 |
| Pelvic Drop | 50 | 0.94 | 0.72 |
| Pelvic Drop | 75 | 0.92 | 0.89 |
| Pelvic Drop | 100 | 0.92 | 0.74 |

**Table S2.** P-values for the main effect of Condition and the Group × Condition interaction at each time point during unilateral countermovement jump (uCMJ) landings, from linear mixed-effects models.

| **Joint Angle** | **Time from IC (ms)** | **p (Condition)** | ****p (Group × Condition)**** |
| --- | --- | --- | --- |
| Knee Flexion | 0 | 0.14 | 0.39 |
| Knee Flexion | 25 | 0.13 | 0.65 |
| Knee Flexion | 50 | 0.31 | 0.80 |
| Knee Flexion | 75 | 0.26 | 0.79 |
| Knee Flexion | 100 | 0.07 | 0.29 |
| Knee Rotation | 0 | 0.44 | 0.94 |
| Knee Rotation | 25 | 0.93 | 0.84 |
| Knee Rotation | 50 | 0.96 | 0.65 |
| Knee Rotation | 75 | 0.54 | 0.65 |
| Knee Rotation | 100 | 0.40 | 0.56 |
| Ankle Dorsiflexion | 0 | 0.68 | 0.17 |
| Ankle Dorsiflexion | 25 | 0.95 | 0.38 |
| Ankle Dorsiflexion | 50 | 0.79 | 0.59 |
| Ankle Dorsiflexion | 75 | 0.92 | 0.84 |
| Ankle Dorsiflexion | 100 | 0.39 | 0.20 |
| Knee Adduction | 0 | 0.19 | 0.32 |
| Knee Adduction | 25 | 0.59 | 0.20 |
| Knee Adduction | 50 | 0.57 | 0.28 |
| Knee Adduction | 75 | 0.13 | 0.17 |
| Knee Adduction | 100 | 0.79 | 0.67 |
| Pelvic Drop | 0 | 0.92 | 0.77 |
| Pelvic Drop | 25 | 0.99 | 0.47 |
| Pelvic Drop | 50 | 0.89 | 0.23 |
| Pelvic Drop | 75 | 0.80 | 0.27 |
| Pelvic Drop | 100 | 0.58 | 0.33 |
